# A U508C synonymous mutation in the SARS-CoV-2 deletion hotspot reduces deletion frequency and accelerates viral clearance

**DOI:** 10.1128/mbio.01984-25

**Published:** 2025-07-31

**Authors:** Kaya Miyazaki, Akihiro Doi, Yuriko Tomita, Wataru Kamitani, Shutoku Matsuyama

**Affiliations:** 1 Department of Respiratory Viruses, National Institute of Infectious Diseases, Tokyo, Japan; 2 Department of Infectious Diseases and Host Defense, Gunma University Graduate School of Medicine, Gunma, Japan; Griffith University, Gold Coast, Queensland, Australia

**Keywords:** SARS-CoV-2, NSP1, deletion, RNA structure

## Abstract

A deletion hotspot within the non-structural protein 1 (NSP1) gene (locus 500–532) has been observed in severe acute respiratory syndrome coronavirus 2 (SARS-CoV-2) genomes analyzed by next-generation sequencing. Deletions in the hotspot have been previously reported to suppress interferon-β expression, suggesting that they constitute a conserved mechanism for SARS-CoV-2 evasion of the immune response. In this study, we introduced a synonymous mutation (U508C) into the SARS-CoV-2 genome using a reverse genetics system. This mutation disrupts both direct and inverted repeat motifs within the deletion hotspot, resulting in a significant reduction in deletion frequency. Furthermore, the clinically isolated U508C mutant strain also exhibited a markedly reduced frequency of deletions compared with the closely related strain from the same lineage, similar to the mutants we generated by reverse genetics. Structural predictions revealed that the wild-type RNA in this region forms a loosely unwound structure, while the RNA with the U508C mutation adopts a more stable structure due to the formation of nine intra-strand base pairs. This increased stability likely reduces polymerase slippage, a major contributor to deletions. Although the U508C mutant replicated as well as that of the wild-type virus in human bronchial epithelial Calu-3 cells, its clearance from the culture supernatant was significantly accelerated. This study provides valuable insights into the intricate interplay between viral RNA structure and viral pathogenesis. Understanding how RNA structural modifications influence viral behavior has broad implications for the development of novel antiviral strategies, including RNA-targeted therapeutics and attenuated virus for vaccine development.

**IMPORTANCE:**

This study focuses on a specific region, termed the “deletion hotspot,” in the SARS-CoV-2 genome where deletions frequently occur and enable the virus to evade the immune response. In this study, we found that the introduction of a U508C synonymous mutation within this deletion hotspot significantly reduced the frequency of deletions, probably because it changed the structure of the viral RNA, stabilizing it and thereby reducing replication errors. While the mutant virus replicated at a similar rate to that of the original virus in cultured cells, its rate of clearance from the culture medium was significantly increased, suggesting that the mutation may have weakened the virus. This study underscores the importance of understanding how the structure of viral RNA influences viral behavior. Such knowledge could inform the development of novel antiviral drugs and strategies for combatting viral infections.

## OBSERVATION

SARS-CoV-2, the virus that causes COVID-19, continues to circulate globally, posing ongoing risks of severe respiratory illness and long-term health complications (1). Developing effective vaccines and antiviral therapeutics requires a thorough understanding of how coronaviruses evade the host immune system. A previous study identified a specific region within the SARS-CoV-2 genome that encodes a viral non-structural protein 1 (NSP1) as a hotspot for deletions (2). Analysis of viral genomes using next-generation sequencing (NGS) revealed a significant number of deletions within this hotspot in both viruses in clinical samples and culture supernatant (2–4). A study by Lin et al. showed that deletions within the 500–532 locus of the viral genome significantly suppress the production of interferon-beta (IFN-β) without affecting the rate of virus replication in cultured cells (2). This finding suggests that the virus may have a conserved mechanism that helps it evade the host immune response by producing internally deleted NSP1. Based on these findings, we hypothesized that introducing a specific mutation within this deletion hotspot would reduce the frequency of deletions and impair the virus’s ability to suppress IFN-β production, resulting in viral attenuation.

**Fig 1:**
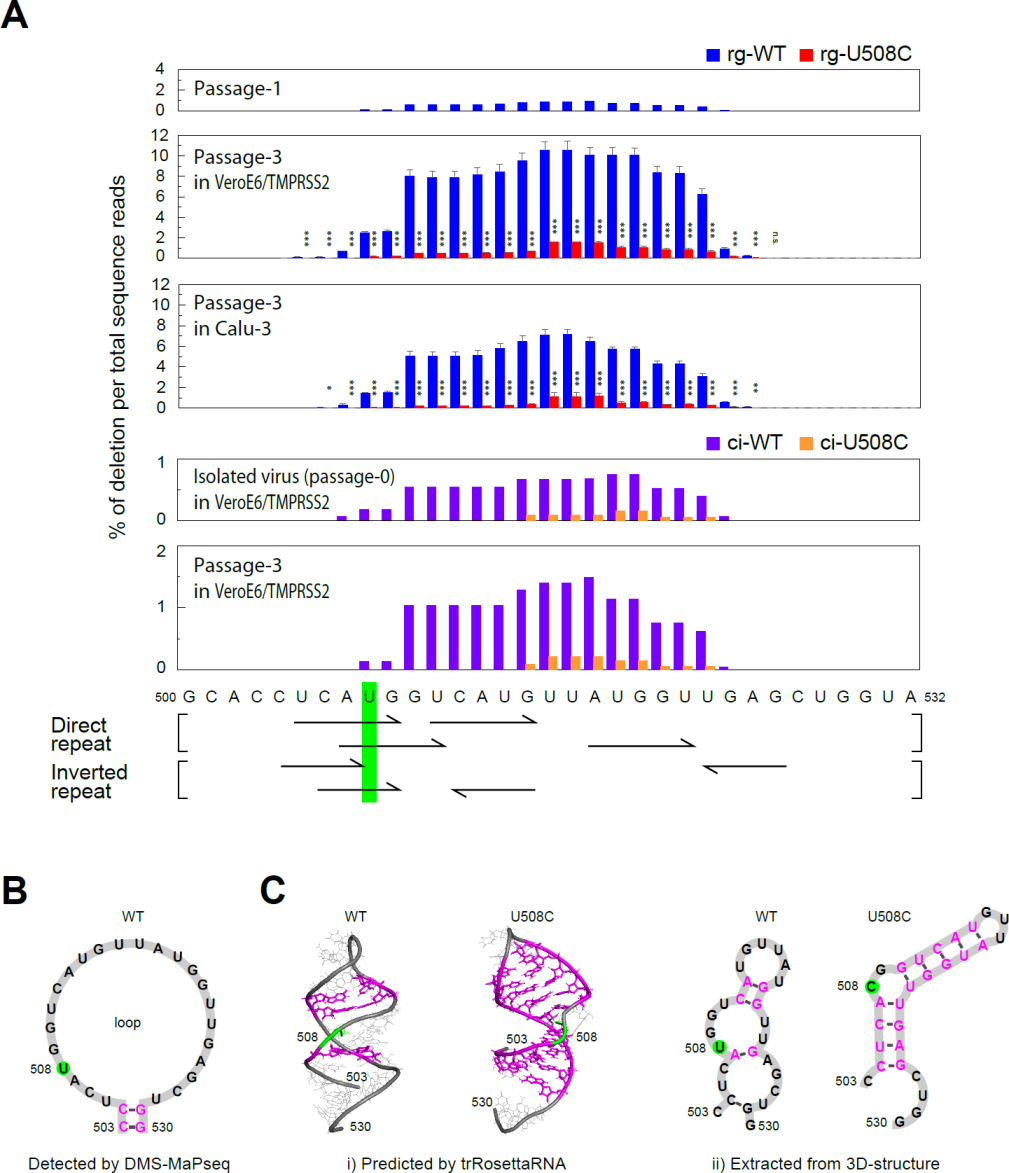
Deletions in the 500–532 locus of the wild-type and U508C mutant SARS-CoV-2 genome. (**A**) percentage of nucleotide deletions in NGS reads. All deletion patterns identified through NGS analysis were aggregated, and the cumulative number of deleted nucleotides at each genomic position was calculated and expressed as a percentage of the total number of sequence reads. Viruses generated by reverse genetics: rg-WT and rg-U508C (number of replicates, *n* = 4); viruses isolated from clinical specimens: ci-WT and ci-U508C (number of replicates, *n* = 1). Two-tailed Student’s *t*-tests were used to analyze the significance of differences between wild types and U508C mutants (n.s., not significant; *, significant [*P* ≤ 0.05]; **, highly significant [*P* ≤ 0.01]; and ***, very highly significant [*P* ≤ 0.001]). Error bars indicate the standard deviations (*n* = 4). (**B**) Secondary structure of the 503–530 region as reported by Lan et al. (6). (**C**) Predicted RNA structures generated using trRosettaRNA, a deep learning–based automated prediction tool. Paired bases are shown in magenta. The “U” highlighted in green represents the nucleotide present in the wild-type virus, which is replaced with a “C” in the U508C mutant.

The deletion hotspot (locus 500–532) in the NSP1 coding region of the SARS-CoV-2 genome contains four consensus motifs, including two direct repeats and two inverted repeats (Fig. 1A), which are known to promote deletions via polymerase slippage (5). Introducing a mutation at position A507 in the viral genome appears to represent an ideal experimental strategy since it would disrupt all four repeat motifs within the deletion hotspot; however, this mutation also results in an amino acid substitution. Thus, we chose the U508C mutation—which disrupts three of the repeat motifs without altering the amino acid sequence—as a more suitable candidate (Fig. 1A). Before introducing the mutation into the virus, we searched to determine whether the U508C mutation is present in circulating viruses. As shown in Fig. S1, search of sequences deposited in the GISAID EpiCoV database confirmed that this mutation has sporadically and naturally arisen in the human population.

Using a BAC plasmid-based reverse genetics system (7), we introduced the U508C mutation into an ancestral SARS-CoV-2 strain (hCoV-19/Japan/TY-WK-521/2020). Following transfection of the BAC plasmid into BHK cells, we recovered four clones: two wild-type and two U508C mutants, which we then used to infect VeroE6/TMPRSS2 cells in independent culture wells (8). Two passages of the viruses were performed in VeroE6/TMPRSS2 cells. In addition, second and third passages were performed using Calu-3 cells, which are derived from the human respiratory epithelium (9). Viral RNA was extracted from the culture supernatant, and the sequences of the NSP1 region were analyzed by NGS. Viral titers and RNA copy numbers at each passage are shown in Fig. S3. Because the infectivity of SARS-CoV-2 in Calu-3 cells is approximately 1/100 of that in VeroE6/TMPRSS2 cells (Fig. S2), it was not possible to infect Calu-3 cells at a high multiplicity of infection (MOI), and a decreasing trend in viral titers was observed with each passage (Fig. S3).

At the 503–530 locus of the SARS-CoV-2 genome, the percentage of each nucleotide deletion was calculated based on total sequence reads, which ranged from 40,000 to 60,000. In the initial passage, the frequency of deletions in the viral genomes obtained through BAC plasmid transfection was extremely low, with deletions occurring at a frequency of less than 1%. The wild-type virus exhibited a slightly higher deletion rate than the U508C mutant virus (Fig. 1A). In the third passage, the deletion rate of the wild-type virus increased, particularly at the 510–523 locus, reaching approximately 10% in the culture supernatant of VeroE6/TMPRSS2 cells and 6% in that of Calu-3 cells. In both cell types, the overall pattern of viral deletions was largely similar (Fig. 1A; Fig. S4). By contrast, deletions in the U508C mutants remained consistently low, with rates below 1.3% (Fig. 1A). By contrast, the frequency of deletions across most patterns in the U508C mutant was markedly lower than that in the wild type (Fig. S4). The most frequent deletion was an out-of-frame deletion of 14 nucleotides, accounting for more than 3.5% of total genome reads (Fig. S4). Viruses harboring out-of-frame deletions are presumably replication-incompetent due to the loss of essential NSPs encoded by ORF1a. It is possible that the defective replication of these viruses is complemented by the replication machinery of co-infecting viruses in the same cells.

Next, we performed the same set of experiments using a clinical isolate harboring the U508C mutation. From the virus strains available in our laboratory, we identified strain EIS01-512, which harbors the U508C mutation, and strain EIS01-529, which belongs to the same lineage but lacks the mutation; both strains have been registered in the GISAID EpiCoV database. These viruses were used to infect VeroE6/TMPRSS2 cells and were serially passaged three times. NGS analysis revealed that the U508C mutant exhibited a lower frequency of deletions than the non-U508C strain, with a deletion pattern similar to that observed in the reverse genetics-derived virus (Fig. 1A).

Lan et al. reported the base pairing of viral RNA in SARS-CoV-2-infected cells using the chemical probe dimethyl sulfate (DMS), which enabled the prediction of the two-dimensional structure of the SARS-CoV-2 genome (6). Their analysis indicated that the RNA at the 503–530 locus forms a loop that does not engage in inter-strand base pairing (as shown in Fig. 1B) (6). To investigate why the single mutation (U508C) within this loop reduces the frequency of most deletion patterns, we attempted to predict the three-dimensional RNA structure of the loop using “trRosettaRNA,” an automated deep learning-based method for the prediction of RNA 3D structure (10). In the wild-type virus, only three bases were predicted to form base pairs in the loop, resulting in an unstable structure resembling the two-dimensional structure identified by Lan et al. (6). By contrast, the U508C mutation led to base pairing involving nine nucleotides, suggesting that it results in a highly stable structure (Fig. 1C; Video S1). This model suggests that bases at the 503–530 locus with the U508C mutation remain paired until the region is unwound by the helicase within the replication-transcription complex, which would potentially reduce polymerase slippage. By contrast, the wild-type virus maintains an unstable loop structure, leading to a higher frequency of deletions.

**Fig 2:**
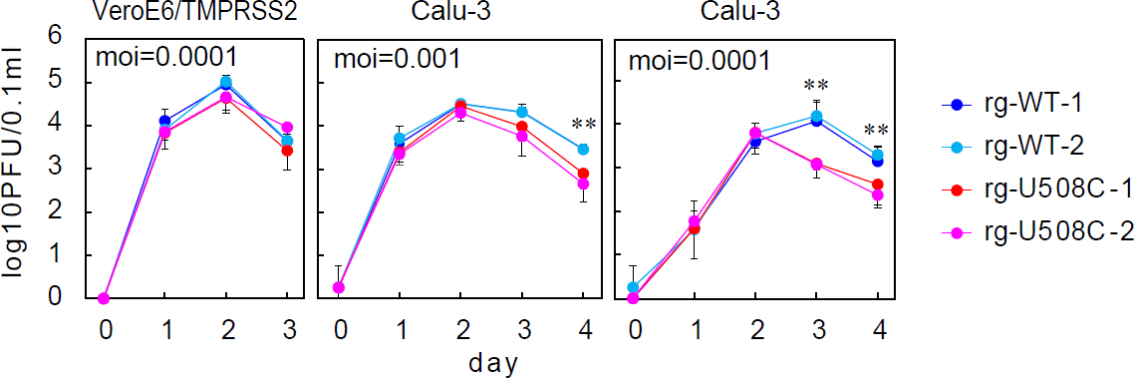
Growth kinetics of SARS-CoV-2 in cultured cells. Infectious virus titers in the culture supernatant of VeroE6/TMPRSS2 or Calu-3 cells following SARS-CoV-2 infection were quantified by the plaque assay using VeroE6/TMPRSS2 cells. Viruses generated by reverse genetics: rg-WT and rg-U508C. Two-tailed Student’s *t*-tests were used to analyze the significance of differences between wild types and U508C mutants (**, highly significant [*P* ≤ 0.01]). Error bars indicate the standard deviations (*n* = 4).

We then investigated the growth characteristics of both wild-type and U508C mutant viruses in cultured cells. In VeroE6/TMPRSS2 cells, four independent viral clones (two wild types and two U508C mutants) exhibited similar growth patterns over the initial two days, followed by a comparable decline in viral titer on day 3 (Fig. 2 left). In Calu-3 cells, both viruses also displayed comparable growth kinetics during the first 2 days of infection while most of the cells remained viable, as shown in Fig. S2. However, starting from day 3, the culture supernatant of the U508C virus exhibited a slightly faster decline in infectious titer compared with the wild-type virus (Fig. 2 center). This difference became more pronounced when the viruses were inoculated at lower multiplicities of infection, resulting in a significantly faster decline for the U508C virus (Fig. 2 right). These observations suggest that, in Calu-3 cells, antiviral factors are gradually induced by the surviving cells as viral replication slowly reaches a plateau, subsequently influencing the decline in viral titers. Under these experimental conditions, viral clearance is expected to be more pronounced at a low MOI. By contrast, Vero-derived cells are known to lack type I interferon genes and therefore cannot mount effective antiviral responses (11), allowing SARS-CoV-2 to replicate rapidly and leading to cell death.

Previous studies by Lin et al. demonstrated that cells infected with SARS-CoV-2 mutants harboring deletions within the 500–532 locus (Δ518–520, Δ510–518, and Δ500–532) exhibited a significant reduction in IFN-β expression (2). To investigate the impact of the U508C mutation on IFN-β production, we infected Calu-3 cells with either the wild-type or U508C mutant virus and quantified IFN-β RNA expression using real-time PCR. However, no significant difference in IFN-β expression was observed between cells infected with wild-type or U508C mutant virus at the time points examined (Fig. S5). This could be because 10% of wild-type viruses have deletions; therefore, the remaining 90% of non-deleted viruses likely exhibit behavior similar to that of the U508C mutant, masking the effects of the deletion-containing subpopulation. At this time, cellular factors responsible for the accelerated clearance of the U508C mutant remain unidentified. We anticipate that prolonged infection with the wild-type virus, which contains a higher proportion of deletion mutations, would lead to lower IFN-β expression than the U508C mutant, as previously demonstrated by Lin et al. (2).

This study shows that a synonymous mutation in SARS-CoV-2 reduces the frequency of deletions. Further systematic research involving the introduction of various mutations into viral RNA to investigate the relationship between RNA structure and viral function could significantly advance the field of RNA structural biology. This, in turn, could accelerate the development of antiviral drugs targeting viral RNA and furnish new strategies for attenuating virus infection.

## Data Availability

A comprehensive description of materials and methods is available in the Supplemental material.

## SUPPLEMENTAL MATERIAL

Supplemental Figures mbio.01984-25-s0001.docx Fig. S1 to S5.

Supplemental Text mbio.01984-25-s0002.docx Supplemental materials and methods.

Video S1 mbio.01984-25-s0003.mpg RNA structure.

ASM does not own the copyrights to Supplemental Material that may be linked to, or accessed through, an article. The authors have granted ASM a non-exclusive, world-wide license to publish the Supplemental Material files. Please contact the corresponding author directly for reuse.
