# Supplementary material for: A U508C synonymous mutation in the SARS-CoV-2 deletion hotspot reduces deletion frequency and accelerates viral clearance
Source: mBio. 2025 Jul 31;16(9):e01984-25. doi: 10.1128/mbio.01984-25 (PMC12421834; doi:10.1128/mbio.01984-25)
Supplement: Supplemental Figures — Fig. S1 to S5. [file mbio.01984-25-s0001.docx]

**Figure S1. Occurrence of the top 20 SARS-CoV-2 lineages harboring the U508C mutation**

Data were extracted from the GISAID EpiCoV database (https://www.gisaid.org). The number of U508C-containing sequences deposited in the database was counted monthly for each viral lineage. The main countries in which each cluster emerged are indicated.

As of April 2025, among approximately 17 million sequences deposited in the GISAID EpiCoV database, 2,689 viral sequences containing the U508C mutation were identified. The distribution of the top 20 viral lineages harboring the U508C mutation is shown. The U508C mutation has appeared sporadically across multiple lineages and geographic regions, forming small, localized clusters. For example, in Canada, between October 2020 and May 2021, 344 sequences with the U508C mutation belonging to the B.1.36 lineage were detected. However, all global occurrences of the U508C mutant subsided within a few months and did not lead to widespread outbreaks. These findings suggest that viruses harboring the U508C mutation arise sporadically and frequently, but are likely to pose a low epidemiological risk.

**Figure S2. Comparison of SARS-CoV-2 infectivity.**

Ten-fold serial dilutions of SARS-CoV-2, either wild-type or U508C mutant, both generated by reverse genetics and passaged twice, were used to infect VeroE6/TMPRSS2 and Calu-3 cells, respectively, in duplicate in 96-well plates. VeroE6/TMPRSS2 cells were cultured for up to 3 days post-infection, and Calu-3 cells were cultured for up to 14 days. After fixation with formalin, cells were stained with crystal violet (left panels). On day 2 post-infection, culture supernatants were collected for plaque assay to determine viral titers (center panels), and viral RNA levels were quantified by real-time PCR (right panels).

In VeroE6/TMPRSS2 cells, cytopathic effects were observed up to approximately the sixth dilution for both viruses, suggesting that the inoculum contained approximately 10⁶ infectious units, and the viral titers and viral RNA levels increases correlated with the extent of cell death. In contrast, Calu-3 cells exhibited only minimal cytopathic effects, the majority of the cells remained viable even after 14 days, and viral replications were detectable only up to the fourth dilution. These findings indicate that the infectivity of SARS-CoV-2 in Calu-3 cells is approximately 1/100 that in VeroE6/TMPRSS2 cells, suggest that high-titer infection of Calu-3 cells with SARS-CoV-2 is not readily achievable.

**Figure S3. Viral titer and RNA copy number of SARS-CoV-2 during serial passages.**

Wild-type (rg-WT) and U508C mutant (rg-U508C) SARS-CoV-2 strains generated by reverse genetics (P0) were initially passaged once in VeroE6/TMPRSS2 cells, followed by two additional passages in either VeroE6/TMPRSS2 or Calu-3 cells. Further, the EIS01-512 strain (ci-WT), which harbors the U508C mutation, and the wild-type EIS01-529 strain (ci-U508C), both viruses possess sequences closely related to lineage XBB.1.5, were isolated from clinical specimens using VeroE6/TMPRSS2 cells (P0) and subsequently passaged three times. At each passage, culture supernatants were collected at 2 days post infection to measure viral titers by plaque assay and viral RNA copy numbers by real-time PCR. The viruses used for the passage in Calu-3 cells were derived from the first passage in the upper panel, therefore, the data for the virus generated by reverse genetics (P0) and the first passage virus (P1) are the same as those used in the upper panel and are indicated by dotted lines. Error bars indicate the standard deviations.

In VeroE6/TMPRSS2 cells, high viral titers were maintained across passages, whereas in Calu-3 cells, the viral titers decreased with each passage. Viral RNA copy numbers were consistently detected at approximately 1,000-fold higher than infectious virus titers. Despite the low infectivity of the P0 virus, a high level of viral RNA was detected, likely reflecting the presence of input BAC plasmid-derived DNA used in the reverse genetics system.

**Figure S4. Top 10 deletion patterns.**

For the virus generated by reverse genetics, the deletion patterns observed within the 503–530 locus of the SARS-CoV-2 genome based on NGS reads are shown in descending order of abundance. For the clinical isolates, the corresponding deletion patterns are presented in the same order as those observed in the reverse genetics-derived virus. The "U" highlighted in green represents the nucleotide present in the wild-type virus, which is replaced with a "C" in the U508C mutant generated by reverse genetics. Two-tailed Student’s t-tests were used to analyze the significance of differences between wild-types and U508C mutants (n.s., not significant; **, highly significant (P≤0.01); and ***, very highly significant (P≤0.001)). Error bars indicate the standard deviations (n=4).

**Figure S5. IFN-β induction.**

Relative expression levels of IFN-β RNA in Calu-3 cells following SARS-CoV-2 infection were quantified using real-time PCR. Two-tailed Student’s t-tests were used to analyze the significance of differences between wild-types and U508C mutants (n.s., not significant). Error bars indicate the standard deviations (n=4).
