# Supplementary material for: A U508C synonymous mutation in the SARS-CoV-2 deletion hotspot reduces deletion frequency and accelerates viral clearance
Source: mBio. 2025 Jul 31;16(9):e01984-25. doi: 10.1128/mbio.01984-25 (PMC12421834; doi:10.1128/mbio.01984-25)
Supplement: Supplemental Text — Supplemental materials and methods. [file mbio.01984-25-s0002.docx]

**SUPPLEMENTAL INFORMATION**

**Materials and Methods**

**Generation of recombinant SARS-CoV-2 from BAC plasmids.**

A BAC clone carrying the full-length infectious genome of the SARS-CoV-2 TY-WK-521/2020 strain, pBAC-SARS2-WK521-wt, was used to generate recombinant SARS-CoV-2, as described previously (1). The BAC infectious clones carrying a U508C mutation were generated by modification of pBAC-SARS2-WK521-wt (as a template) using a counter-selection BAC modification kit employing the Red/ET recombination system (Gene Bridges, Heidelberg, Germany). The sequences of all of the constructs were confirmed using Sanger sequencing methods (Eurofins, Nantes, France). BHK-21 cells were grown in a single well of a 24-well plate in 10% fetal calf serum (FCS)–minimal essential medium (MEM) and transfected with 3 µg BAC plasmid using Polyethylenimine Max (Polysciences, USA). After transfection, Vero/TMPRSS2 cells were inoculated onto transfected BHK-21 cells. The coculture was then incubated at 37°C for 3 days. The supernatants were collected and propagated once in Vero/TMPRSS2 cells. Recovered viruses were stored at -80°C.

**Targeted Next-Generation Sequencing Analysis of SARS-CoV-2 Genome Regions**

A modified version of the Wellcome Trust ARTIC Network protocol (<https://artic.network/ncov-2019>) was used to sequence the nucleotide 343–704 region of the SARS-CoV-2 genome, which was amplified by RT-PCR using region-specific primers (nCoV-2019_2_LEFT-F [CTGTTTACAGGTTCGCGACGT] and nCoV-2019_2_RIGHT-R [TAAGGATCAGTGCCAAGCTCGT]). The obtained RT-PCR amplicons were used as input for library preparation using the QIAseq FX DNA Library Kit (Qiagen) and sequenced on the iSeq 100 or MiSeq platform (Illumina). The resulting NGS reads were mapped to the SARS-CoV-2 Wuhan-Hu-1 reference genome sequence (GenBank ID: MN908947) using CLC Genomics Workbench version 22 (Qiagen), resulting in an average of 302,425 reads.

**RNA Structure Prediction**

The 3D RNA structures of the 503–532 locus in the SARS-CoV-2 genome, with and without the U508C mutation, were predicted using an automated deep learning-based method, trRosettaRNA (2), which is available online at https://yanglab.qd.sdu.edu.cn/trRosettaRNA/.

**Quantification of Viral Replication and IFN-β Expression**

Human bronchial epithelial Calu-3 cells and VeroE6/TMPRSS2 cells were seeded in 96-well plates and infected with wild-type or U508C mutant SARS-CoV-2 at a multiplicity of infection (MOI) of 0.001 or 0.0001. Following incubation for up to 4 days, the culture medium was collected, and viral titers were quantified using the plaque assay with VeroE6/TMPRSS2 cells (3). To assess SARS-CoV-2 viral RNA levels in culture supernatants, RNA was extracted with ISOGEN-LS reagent (317-02623; Nippon Gene). Quantitative real-time PCR was conducted on a MyGo Pro instrument (IT-IS Life Science, Ireland) using the following primers and probe: E_Sarbeco_F (ACAGGTACGTTAATAGTTAATAGCGT), E_Sarbeco_R ( ATATTGCAGCAGTACGCACACA), and E_Sarbeco_P1-FAM (ACACTAGCCATCCTTACTGCGCTTCG) (4). To assess IFN-β mRNA levels, total cellular RNA was extracted with ISOGEN reagent (311-02501; Nippon Gene). Quantitative real-time PCR was conducted using the following primers and probe: hIFN-β-F (AAACTCATGAGCAGTCTGCA), hIFN-β-R (AGGAGATCTTCAGTTTCGGAGG), and hIFN-β-FAM probe (ATGGTCCAGGCACAGTGACTGTCCTC) (5).

**Statistical analysis**

Statistical significance was assessed using a two-tailed Student t test. A P value of <0.05 was considered statistically significant. In figures, significance is indicated as follows: n.s., not significant; *, significant (P ≤ 0.05); **, highly significant (P ≤ 0.01); and ***, very highly significant (P ≤ 0.001). Error bars indicate standard deviations (SD).

**References**

1. Ueno S, Amarbayasgalan S, Sugiura Y, Takahashi T, Shimizu K, Nakagawa K, Kawabata-Iwakawa R, Kamitani W. 2024. Eight-amino-acid sequence at the N-terminus of SARS-CoV-2 nsp1 is involved in stabilizing viral genome replication. Virology 595:110068.

2. Wang W, Feng C, Han R, Wang Z, Ye L, Du Z, Wei H, Zhang F, Peng Z, Yang J. 2023. trRosettaRNA: automated prediction of RNA 3D structure with transformer network. Nat Commun 14:7266.

3. Matsuyama S, Nao N, Shirato K, Kawase M, Saito S, Takayama I, Nagata N, Sekizuka T, Katoh H, Kato F, Sakata M, Tahara M, Kutsuna S, Ohmagari N, Kuroda M, Suzuki T, Kageyama T, Takeda M. 2020. Enhanced isolation of SARS-CoV-2 by TMPRSS2-expressing cells. Proc Natl Acad Sci USA 117:7001–7003.

4. Corman VM, Landt O, Kaiser M, Molenkamp R, Meijer A, Chu DK, Bleicker T, Brünink S, Schneider J, Schmidt ML, Mulders DG, Haagmans BL, van der Veer B, van den Brink S, Wijsman L, Goderski G, Romette JL, Ellis J, Zambon M, Peiris M, Goossens H, Reusken C, Koopmans MP, Drosten C. 2020. Detection of 2019 novel coronavirus (2019-nCoV) by real-time RT-PCR. Euro Surveill 25:1–8.

5. Jaworska J, Gravel A, Fink K, Grandvaux N, Flamand L. 2007. Inhibition of Transcription of the Beta Interferon Gene by the Human Herpesvirus 6 Immediate-Early 1 Protein. J Virol 81:5737–5748.
